# TMC4 localizes to multiple taste cell types in the mouse taste papillae

**DOI:** 10.1002/2211-5463.70159

**Published:** 2025-11-11

**Authors:** Momo Murata, Yoshikazu Saito, Yoichi Kasahara, Wataru Fujii, Takumi Misaka, Tomiko Asakura, Masataka Narukawa

**Affiliations:** ^1^ Graduate School of Home Economics Kyoto Women's University Kyoto Japan; ^2^ Research Department Toyo Institute of Food Technology Hyogo Japan; ^3^ Department of Applied Biological Chemistry, Graduate School of Agricultural and Life Sciences The University of Tokyo Tokyo Japan; ^4^ Department of Veterinary Medical Sciences, Graduate School of Agricultural and Life Sciences The University of Tokyo Tokyo Japan; ^5^ Department of Liberal Arts The Open University of Japan Chiba Japan; ^6^ Department of Food and Nutrition Kyoto Women's University Kyoto Japan

**Keywords:** amiloride‐insensitive salty taste, circumvallate papillae, fungiform papillae, taste cell type, TMC4

## Abstract

Salty taste is mediated through two distinct transduction pathways: the amiloride‐sensitive and amiloride‐insensitive (AI). Transmembrane channel‐like 4 (TMC4), a voltage‐dependent chloride channel, has recently been identified as playing a critical role in AI‐mediated salt taste responses. Although its functional properties have been demonstrated, the specific taste cell types that express TMC4 have not been fully characterized. To investigate the cellular localization of TMC4, we generated *Tmc4*‐EGFP knock‐in mice and performed immunohistochemical analyses of the taste papillae. By performing multiple fluorescent immunostaining with cell‐type‐specific markers, such as KCNQ1 (type I–III), Gustducin and PLCβ2 (type II), and AADC (type III), together with EGFP, we found that over 98% of EGFP‐positive cells overlapped with the signal of all three taste cell type markers in the circumvallate papillae of *Tmc4*‐EGFP mice. In the fungiform papillae, 95.9% of EGFP‐positive cells colocalized with KCNQ1. These results demonstrate that TMC4 is broadly expressed across all three taste cell types, and suggest the possible involvement of multiple, functionally distinct taste cell populations in AI‐mediated salty taste transduction.

## Graphical abstract

Transmembrane channel‐like 4 (TMC4), a voltage‐dependent chloride channel, plays a critical role in amiloride‐insensitive salty taste transduction. TMC4 is broadly expressed in all mature taste cell types, suggesting a possible involvement of multiple cell types in this pathway.

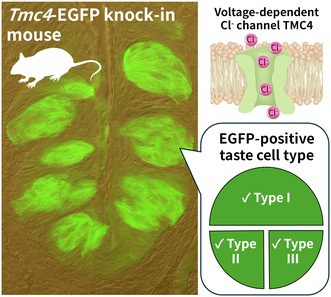

**Abbreviations**
AADC: aromatic l‐amino acid decarboxylase
AI: amiloride‐insensitive
AS: amiloride‐sensitive
CvP: circumvallate papillae
DAPI: 4'6‐diamidino‐2‐phenylindole
DIC: differential interference contrast
EGFP: enhanced green fluorescent protein
ENaC: epithelial sodium channel
FuP: fungiform papillae
hCG: human chorionic gonadotropin
IHC: immunohistochemistry
ISH: *in situ* hybridization
PBS: phosphate‐buffered saline
PFA: paraformaldehyde
PMSG: pregnant mare serum gonadotropin
rGpA: rabbit globin polyadenylation signal
TMC4: transmembrane channel‐like 4

The sense of taste enables animals to evaluate the nutritional value and safety of foods before ingestion. It comprises five fundamental modalities: sweet, umami, bitter, sour, and salty. Saltiness represents the detection of essential electrolytes, primarily sodium, which is vital for maintaining fluid homeostasis and supporting electrical activity in excitable tissues [1, 2]. Saltiness exhibits different characteristics depending on the concentration, eliciting preference at low concentrations and aversion at high concentrations.

Taste detection begins at the level of taste buds, which are multicellular structures embedded within specialized papillae on the tongue surface. Each taste bud contains approximately 50–150 taste cells and is located predominantly within the fungiform (FuP), foliate, and circumvallate papillae (CvP) [1, 2]. These papillae differ in location and innervation: the anterior FuP are supplied by the chorda tympani nerve, whereas the posterior foliate and CvP are innervated by the glossopharyngeal nerve. These afferent pathways convey taste information to the central nervous system [3].

Taste bud cells are classified into four main types (I–IV), each defined by its specific structural and molecular features [4]. Types I–III are considered mature taste cells, with type II and III cells primarily responsible for taste perception. Type II cells are responsible for detecting sweet, bitter, and umami stimuli, whereas type III cells mediate responses to sour taste. Although the identity of the specific cell populations responsible for mediating salty taste perception remains controversial, part of the salty taste relies on a subpopulation of the bitter‐responsive type II cells and a subset of the sour‐responsive type III cells [5, 6]. Some studies have also suggested that type I taste cells are associated with salty taste detection [7]. Type I cells are thought to serve supportive and homeostatic functions within the taste buds, somewhat akin to glial cells in the brain. Although they comprise nearly half of all taste cells, their roles in direct chemosensation remain poorly characterized. However, a recent study has indicated that type I cells may mediate intercellular mechanisms, such as sweet taste adaptation [8]. Type IV cells act as basal progenitors, giving rise to three other cell types during turnover.

Salty taste is mediated through two distinct transduction pathways, defined by sensitivity to the diuretic amiloride: the amiloride‐sensitive (AS) and amiloride‐insensitive (AI) [9]. The AS pathway, predominant at low NaCl concentrations, involves the epithelial sodium channel (ENaC) and is blocked by amiloride [5]. In contrast, the AI pathway is engaged at higher salt concentrations and is predominantly transmitted via the glossopharyngeal nerve [5, 10].

Despite considerable research, the molecular identity of the AI pathway remains elusive until recently. We previously identified transmembrane channel‐like 4 (TMC4) as a voltage‐dependent chloride channel that plays a critical role in AI‐mediated salt taste responses [11]. TMC4 mediates the permeation of chloride and various organic anions and is inhibited by the anion channel blocker, NPPB. *Tmc4* mRNA signals were observed specifically in taste buds in the mouse oral cavity and were stronger in CvP than in FuP. Furthermore, *Tmc4*‐deficient mice showed markedly reduced glossopharyngeal nerve responses to high concentrations of NaCl, confirming its physiological relevance.

While the functional role of TMC4 has been demonstrated, the precise taste cell types in which TMC4 is expressed remain unknown. In this study, to gain further insight into the role of TMC4 in AI salt taste transduction, we generated *Tmc4*‐enhanced green fluorescent protein (EGFP) knock‐in mice using the CRISPR‐Cas9 system, introducing an EGFP reporter downstream of the *Tmc4* stop codon. Although this knock‐in model does not reflect the precise expression level of *Tmc4* mRNA, it is a useful tool for visualizing the spatial distribution of its expression. We investigated the cellular localization of TMC4 in the taste papillae of *Tmc4*‐EGFP mice.

## Methods

### Animals

*Tmc4*‐EGFP mice were generated with support from the Research Support Project for Life Science and Drug Discovery (Basis for Supporting Innovative Drug Discovery and Life Science Research, BINDS).

C57BL/6J mice were purchased from Jackson Laboratory Japan (Yokohama, Japan). Mice were maintained in plastic cages under pathogen‐free conditions in a room at 23.5 ± 2.5 °C and 52.5% ± 12.5% relative humidity under a 14‐h light:10‐h dark cycle. Mice had free access to commercial chow (MF; Oriental Yeast Co., Ltd., Tokyo, Japan) and filtered water. Animal experiments were conducted in a humane manner with approval from the Institutional Animal Experiment Committee of the University of Tsukuba, in accordance with the Regulations for Animal Experiments of the University of Tsukuba and the Fundamental Guidelines for Proper Conduct of Animal Experiments and Related Activities in Academic Research Institutions under the jurisdiction of the Ministry of Education, Culture, Sports, Science, and Technology of Japan. Heterozygous mice were introduced into animal facilities at the University of Tokyo and Kyoto Women's University and intercrossed to generate homozygous offspring. All experiments were performed under protocols approved by the Kyoto Women's University Animal Care Committee (Approval numbers: 2024‐15 and 2025‐8) and the University of Tokyo Animal Care Committee (Approval Number: P24‐125).

### Preparation of CRISPR components and vector construction

We selected a sequence (5′‐GTG ACT TCA TAT CAA GTG AT‐3′) downstream of the termination codon of *Tmc4* as the CRISPR target. Synthetic crRNA containing this sequence and tracrRNA were purchased from IDT (Coralville, IA, USA). TrueCut Cas9 Protein v2 was purchased from Thermo Fisher Scientific (Waltham, MA, USA).

In the *pTmc4‐IRES‐EGFP* donor DNA, we placed the *IRES*‐*EGFP*‐*rabbit globin polyadenylation signal* (*rGpA*) sequence between the 5′ and 3′ homology arms. The 5′‐homology arm spanned 1146 bp upstream and 51 bp downstream of the *Tmc4* stop codon, and the 3′‐homology arm spanned 52–1251 bp downstream of the stop codon. This donor DNA plasmid vector was isolated with the FastGene Plasmid Mini Kit (Nippon Genetics, Tokyo, Japan) and filtered using a MILLEX‐GV® 0.22 μm filter unit (Merck Millipore, Darmstadt, Germany) for microinjection.

### Microinjection

After superovulation with pregnant mare serum gonadotropin (PMSG) and human chorionic gonadotropin (hCG), female C57BL/6J mice were mated with male mice, and zygotes were obtained. The CRISPR‐Cas9 ribonucleoprotein complex and donor DNA were microinjected into zygotes according to a previous report [12]. Injected embryos were transferred into pseudopregnant ICR mice.

### Genomic PCR and sequence analysis

To confirm the knock‐in allele, genomic DNA was purified from mouse tails (3‐week‐old) using PI‐200 (KURABO INDUSTRIES LTD., Osaka, Japan) according to the manufacturer's protocol. Genomic PCR was performed with KOD‐Fx (TOYOBO, Osaka, Japan). PCR products were purified with a FastGene Gel/PCR Extraction Kit (Nippon Genetics) and used as templates for sequencing with the BigDye™ Terminator v3.1 Cycle Sequencing Kit (Thermo Fisher Scientific). Sequences were analyzed using a 3500 Series Genetic Analyzer (Thermo Fisher Scientific). We confirmed correct integration of the donor construct at the target locus by sequencing and ruled out random integration using PCR. Although off‐target effects were not assessed in this study, our genotyping approach ensured specific and targeted insertion of the construct.

Genotyping PCR was performed in a 20 μL reaction using KOD One® PCR Master Mix (Toyobo), with genomic DNA, primers (Table S1), and double‐distilled water. Thermal cycling conditions were: 98 °C for 2 min; followed by 30 cycles of 98 °C for 10 s, 60 °C for 5 s, and 68 °C for 5 s; and a final extension at 68 °C for 7 min. PCR products were separated on a 1% agarose gel and visualized under UV light after staining with ethidium bromide.

### Preparation of tissue sections

Male homozygous mice were used for tissue analysis. For immunohistochemistry (IHC) and fluorescence reporter observation, mice were sacrificed by an overdose of intraperitoneal sodium pentobarbital and transcardially perfused with ice‐cold phosphate‐buffered saline (PBS), followed by fixation with 4% paraformaldehyde (PFA) in PBS. The CvP and FuP were dissected, postfixed in 4% PFA/PBS at 4 °C overnight, cryoprotected in 20% sucrose/PBS at 4 °C until they sank, and frozen in O.C.T. compound (Sakura Finetek, Tokyo, Japan). For *in situ* hybridization (ISH), the CvP was dissected from the tongue after cervical dislocation, and it sank and froze in O.C.T. compound. All tissue blocks were stored at −80 °C until use. Tissue blocks were sectioned at 10 μm using a cryostat (Cryostar NX70; Thermo Scientific). Sections were mounted onto MAS‐coated glass slides (Matsunami Glass, Osaka, Japan) and stored at −80 °C until further use.

### Fluorescence reporter analysis

To visualize EGFP signals, CvP sections were immersed in PBS. Fluorescent images were acquired using a BX60 microscope (Olympus, Tokyo, Japan) equipped with an AdvanCam‐E3R digital camera (Advan Vision, Tokyo, Japan).

### Combination of ISH and IHC

The ISH procedure was performed as previously described [11]. Fresh frozen CvP sections were fixed in PBS containing 4% PFA and 0.1% H_2_O_2_ for 30 min at room temperature. The sections were then treated with 1 μg·mL^−1^ proteinase K for 5 min at 30 °C. Slides were acetylated with 0.25% acetic anhydride in 10 mm triethanolamine for 10 min at room temperature. After pre‐hybridization, sections were hybridized with a DIG‐labeled *Tmc4* antisense probe at 58 °C overnight. Post‐hybridization, slides were incubated with peroxidase‐conjugated anti‐DIG antibody (1 : 50; Roche Diagnostics, Basel, Switzerland) for 1 h at room temperature. The TMC4 signal was detected using the TSA system with CF 488A tyramide (final concentration 2 μm; 92 171, Biotium, Fremont, CA, USA) for 10 min at room temperature. For subsequent IHC, slides were washed with PBS, blocked with PBS‐T containing 5% normal horse serum (ab139501, Abcam, Cambridge, UK), and incubated overnight at 4 °C with goat polyclonal anti‐GFP antibody (1 : 500; ab6673, Abcam). Alexa Fluor 555 donkey anti‐goat IgG (1 : 500; A21432, Thermo Fisher Scientific) was applied for 1 h at room temperature. Nuclear staining was performed using 4′,6‐diamidino‐2‐phenylindole (DAPI) (1 μg·mL^−1^) for 30 min. Sections were mounted with Fluoromount‐G (SouthernBiotech, Birmingham, AL, USA) and imaged using an All‐in‐one fluorescence microscope (BZ‐X810; KEYENCE, Osaka, Japan). Images were overlaid using Photoshop Elements 14 (Adobe Systems, San Jose, CA, USA).

### Multiple IHC

Fluorescent IHC was performed as described previously [13, 14, 15]. CvP and FuP sections were washed with PBS and incubated in antigen retrieval solution for 20 min at 80 °C (Dako Target Retrieval Solution, pH 9; Agilent Technologies, Santa Clara, CA, USA).

For double staining, slides were blocked with PBS containing Blocking One Histo (Nacalai Tesque, Kyoto, Japan) and incubated overnight at 4 °C with goat polyclonal anti‐GFP and/or rabbit polyclonal anti‐KCNQ1 (1 : 1000; AB5932, Merck Millipore), rabbit polyclonal anti‐PLCβ2 (1 : 500; sc‐206, Santa Cruz Biotechnology, Dallas, TX, USA), and rabbit polyclonal anti‐aromatic l‐amino acid decarboxylase (AADC) antibodies (1 : 500; GTX134053, GeneTex, Irvine, CA, USA). After PBS washes, Alexa Fluor 488 donkey anti‐goat IgG (1 : 500; A11055, Thermo Fisher Scientific) and Alexa Fluor 555 donkey anti‐rabbit IgG (1 : 500; A31572, Thermo Fisher Scientific) were applied for 1 h at room temperature, followed by DAPI staining (1 μg·mL^−1^, 5 min).

For triple staining, chicken polyclonal anti‐GFP (1 : 500; GFP‐1020, Aves Labs), rabbit polyclonal anti‐PLCβ2, and goat polyclonal anti‐CAR4 (1 : 500; AF2414, R&D Systems, Minneapolis, MN, USA) were used as primary antibodies. Alexa Fluor 488 donkey anti‐chicken IgG (1 : 500; A78948, Thermo Fisher Scientific), Alexa Fluor 406 F(ab’)_2_ donkey anti‐rabbit IgG (1 : 500; SA000010, Thermo Fisher Scientific), and Alexa Fluor 555 donkey anti‐goat IgG were used as secondary antibodies. Sections were mounted with Fluoromount‐G and imaged using a BX60 microscope with AdvanCam‐E3R. Images were processed using Adobe Photoshop Elements 14. To detect clear signals for taste marker proteins, an antigen retrieval step was included. However, because this treatment attenuated EGFP fluorescence, EGFP was visualized using an anti‐GFP antibody in multiple IHC.

The staining samples from three mice were quantitatively analyzed. Cells with immunoreactive signals were counted in three sections per CvP and two sections per FuP (every 50 μm). Taste bud cells were identified from differential interference contrast (DIC) and DAPI images. The percentage of immunoreactive cells was expressed as the number of immunopositive cells per taste bud. Data are expressed as mean ± standard error of the mean.

## Results

### Generation of *Tmc4*‐EGFP mice

**Fig. 1:**
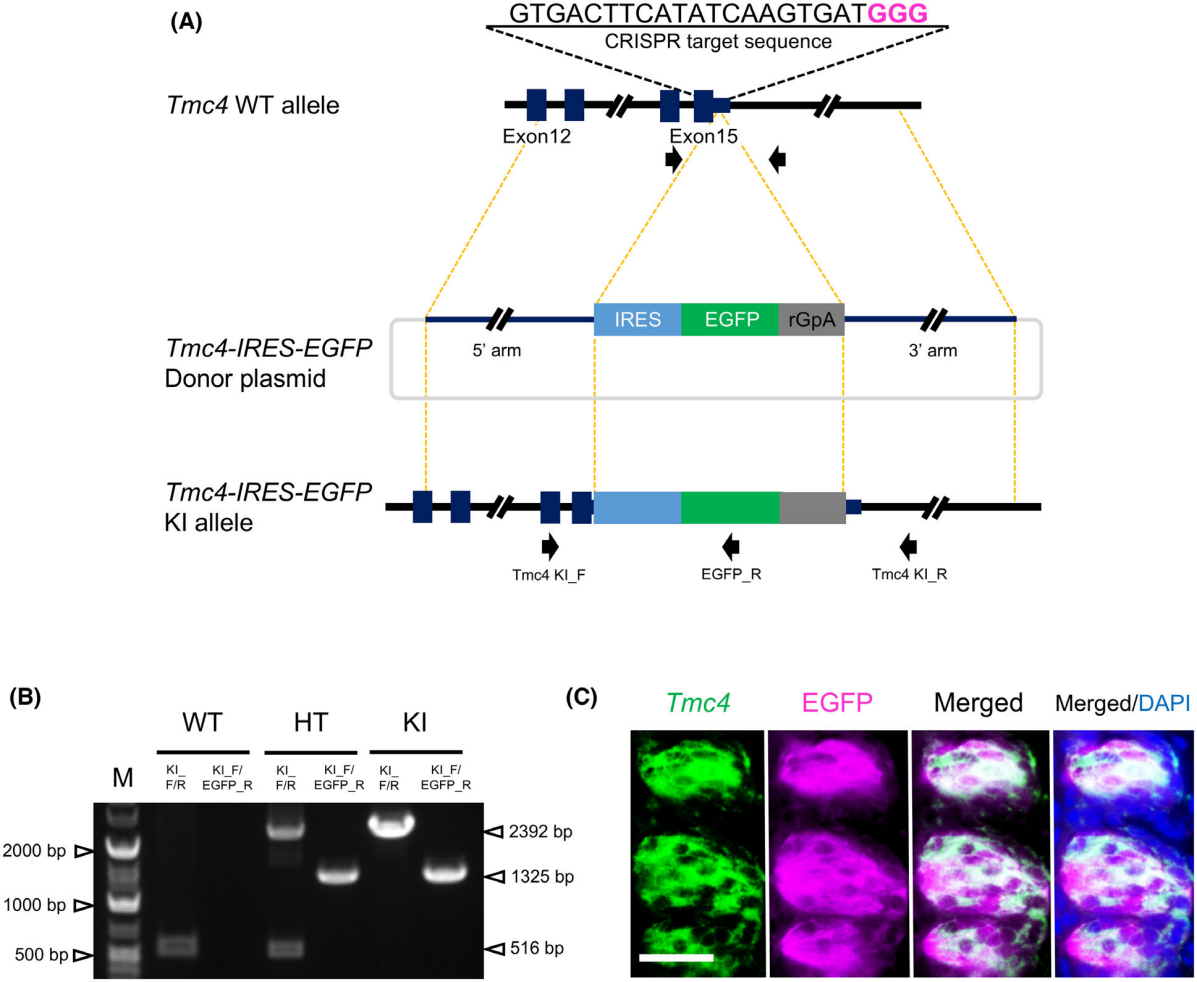
CRISPR design for *Tmc4‐*EGFP mice. (A) Knock‐in strategy for the *Tmc4*‐EGFP allele. The CRISPR target site (underlined) was designed including the protospacer adjacent motif GGG (PAM) (magenta bold). The IRES sequence (sky blue box) was fused with the reporter gene EGFP (yellow‐green) and polyadenylation signal (pA) (gray) and inserted immediately after the stop codon. Primers used for PCR genotyping (Table S1) are indicated by black arrows. (B) Genotyping of homozygous knock‐in mice. Representative gel electrophoresis image showing amplification products for C57BL/6J (WT), heterozygous (HT), and homozygous knock‐in (KI) alleles. Genomic DNA was extracted from tail biopsies. *n* = 1 biologically independent mouse per genotype. (C) Overlap of *Tmc4* mRNA and EGFP protein signals in the circumvallate papilla of *Tmc4*‐EGFP mice. EGFP was detected using an anti‐GFP primary antibody and a fluorescent secondary antibody. Representative image from *n* = 1 biologically independent mouse. Scale bar: 20 μm.

An outline of the CRISPR design is shown in Fig. 1A. EGFP was selected as a fluorescent reporter gene and inserted near the stop codon in exon 15 of the *Tmc4* gene. The knock‐in allele was confirmed by PCR (Fig. 1B), and a homozygous individual was used for subsequent analyses. To confirm the expression of reporter proteins under the control of the endogenous *Tmc4* promoter, *Tmc4*‐EGFP mice were dissected and examined for fluorescent signals. In the CvP of Tmc4‐EGFP mice, EGFP signals were detected (Fig. S1). *Tmc4* mRNA expression overlapped with EGFP protein signals in the CvP of *Tmc4*‐EGFP mice (Fig. 1C). As expected, no hybridization signals were detected using a *Tmc4* sense probe (Fig. S2). These results indicate that *Tmc4*‐EGFP mice successfully labeled TMC4‐expressing cells with EGFP.

### Expression of various taste cell marker molecules in EGFP‐expressing cells

**Fig. 2:**
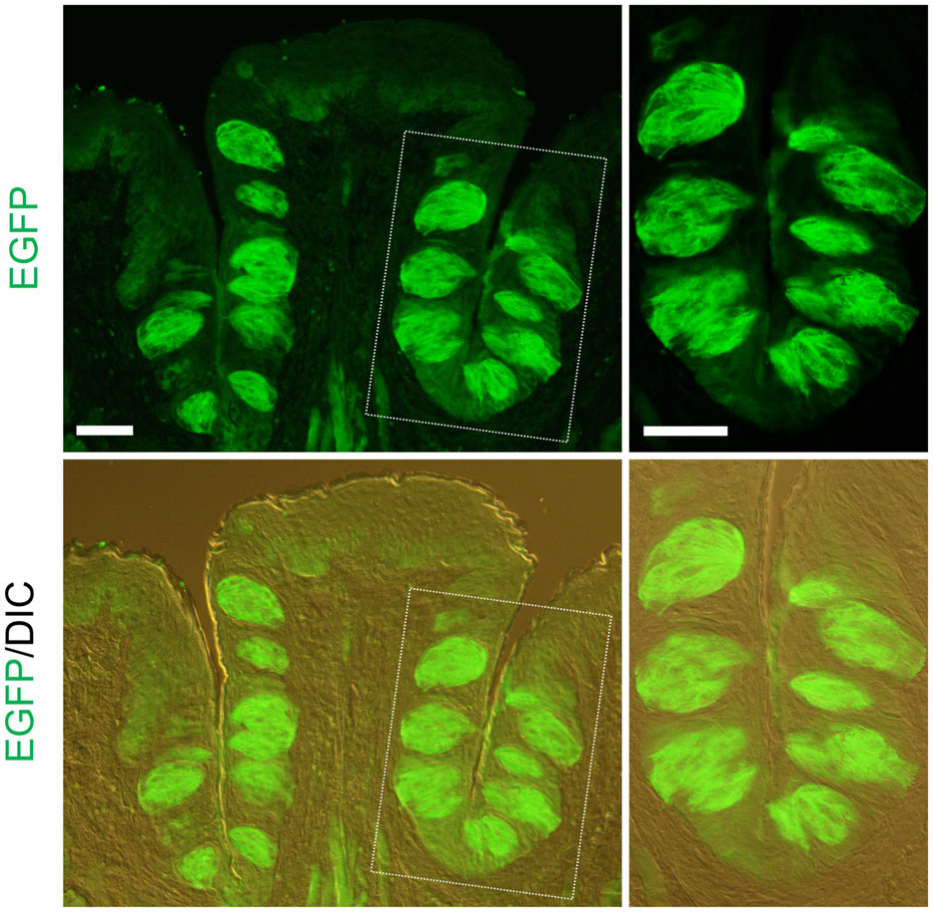
Immunostaining with GFP antibody in the circumvallate papillae of *Tmc4*‐EGFP mice. EGFP signal (green) was visualized using an anti‐GFP primary antibody and a fluorescent secondary antibody. A merged image with differential interference contrast (DIC) is shown. The right panel shows a magnified view of the area enclosed by the dotted line. Representative image from *n* = 1 biologically independent mouse. Scale bars: 50 μm.

We first performed immunostaining of taste papillae using an anti‐GFP antibody. EGFP signals were detected in the CvP of *Tmc4*‐EGFP mice (Fig. 2). However, no EGFP signals were observed in the CvP of C57BL/6J mice (Fig. S3A). In addition, no EGFP signals were detected in the CvP of *Tmc4*‐EGFP mice when the anti‐GFP antibody was omitted (Fig. S3B).

**Fig. 3:**
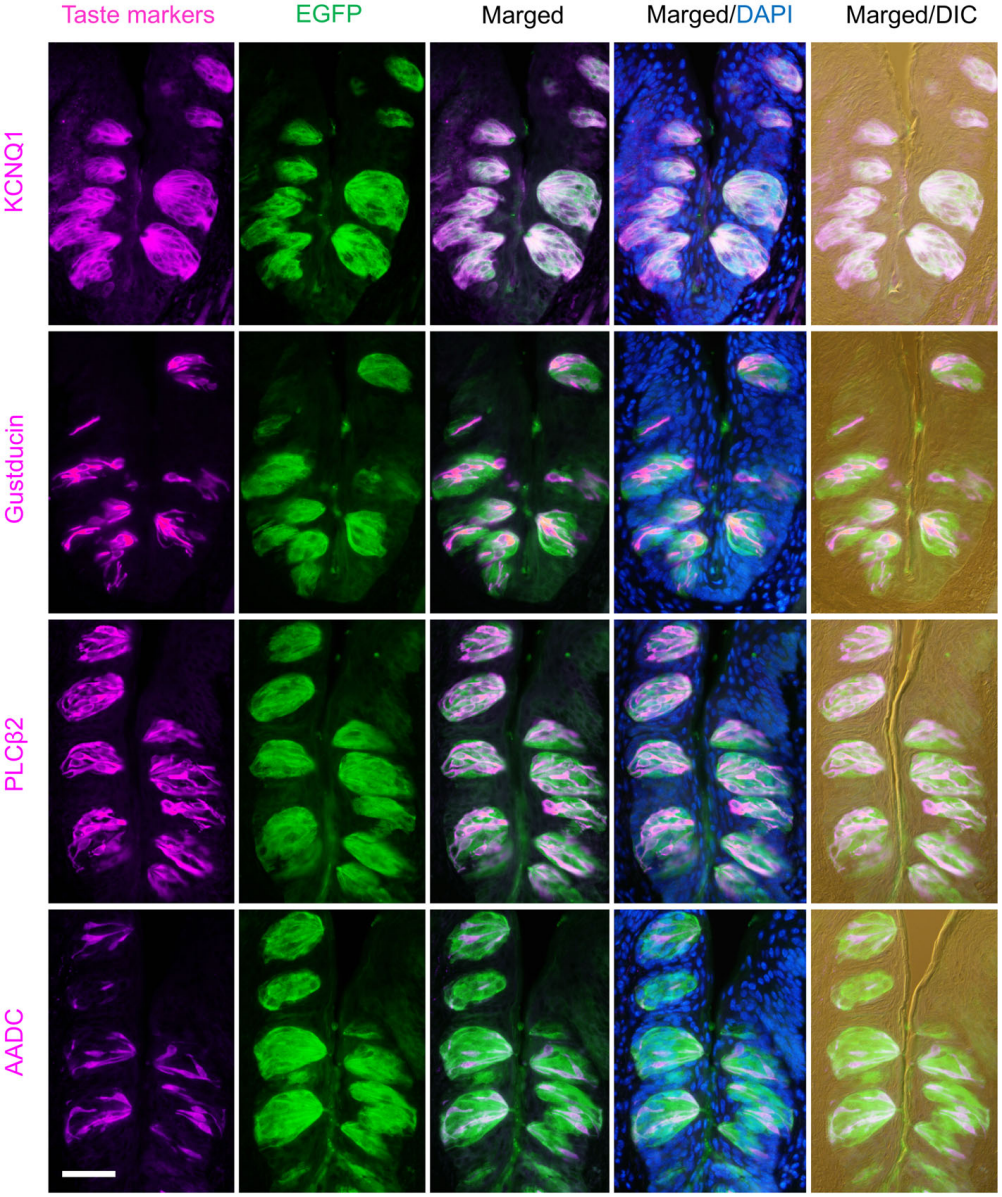
Coexpression of taste cell markers with EGFP in the circumvallate papillae of *Tmc4*‐EGFP mice. Double‐fluorescent immunostaining was performed using antibodies against KCNQ1, Gustducin, PLCβ2, and AADC (magenta), and EGFP (green). Merged images with DAPI nuclear staining (blue) are shown. The rightmost column displays merged images with differential interference contrast (DIC). Representative image from *n* = 1 biologically independent mouse. Scale bar: 50 μm.

Next, to identify the taste cell types that express EGFP, we performed double immunofluorescence staining using antibodies against taste cell markers, including the type I–III marker KCNQ1 [16], the type II markers Gustducin [17] and PLCβ2 [16], and the type III marker AADC [16], along with an anti‐GFP antibody, in the CvP of *Tmc4‐*EGFP mice (Fig. 3). EGFP signals colocalized with each of these marker proteins. Among KCNQ1‐positive cells, 98.5% expressed EGFP (Table 1). Similarly, 99.7% of PLCβ2‐positive cells, 98.4% of gustducin‐positive cells, and 99.3% of AADC‐positive cells expressed EGFP (Table 1).

**Table 1:** The coexpression ratio of EGFP and taste marker genes in the circumvallate papillae. Total number of counted cells was given in parentheses.

	Coexpression ratio (%)			
	KCNQ1	Gustducin	PLCβ2	AADC
EGFP/taste marker gene^a^	98.5% (3824/3883)	98.4% (935/950)	99.7% (1271/1275)	99.3% (820/826)
Taste marker gene/EGFP^b^	98.8% (3824/3872)	22.9% (935/4082)	28.0% (1271/4536)	16.8% (820/4880)

^a^ The coexpression ratio of the cells expressing both EGFP and a taste marker gene to the cells expressing a taste marker gene

^b^ The coexpression ratio of the cells expressing both EGFP and a taste marker gene per the cells expressing EGFP.

**Fig. 4:**
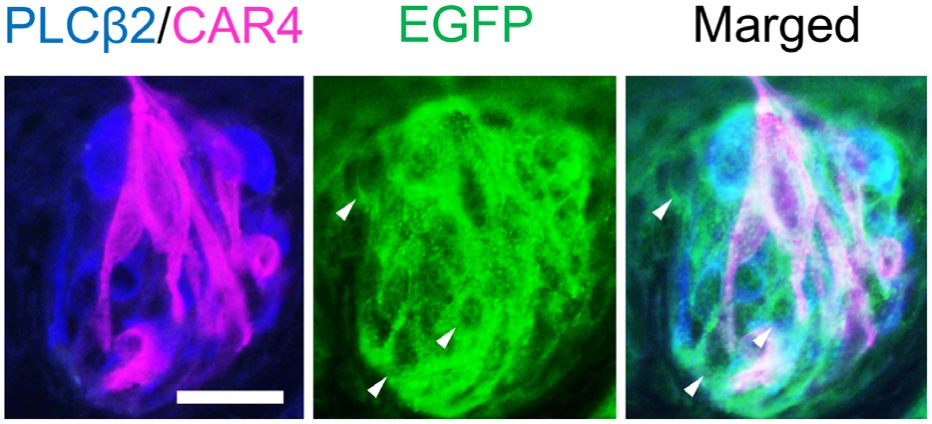
Triple immunostaining of taste cell types in the circumvallate papillae of *Tmc4*‐EGFP mice. Immunostaining was performed using antibodies against EGFP (green), PLCβ2 (blue), and CAR4 (magenta) in the circumvallate papillae of *Tmc4*‐EGFP mice. Arrowheads indicate cells positive for EGFP but negative for PLCβ2 and CAR4. Representative image from *n* = 1 biologically independent mouse. Scale bar: 20 μm.

To further characterize EGFP‐positive cells, we performed triple immunofluorescence staining using antibodies against GFP, PLCβ2, and the type III marker CAR4 [16]. We observed EGFP‐positive cells that expressed neither PLCβ2 nor CAR4, indicating that EGFP is also expressed in type I cells (Fig. 4). These results suggest that TMC4 is expressed in type I, II, and III taste cells.

**Fig. 5:**
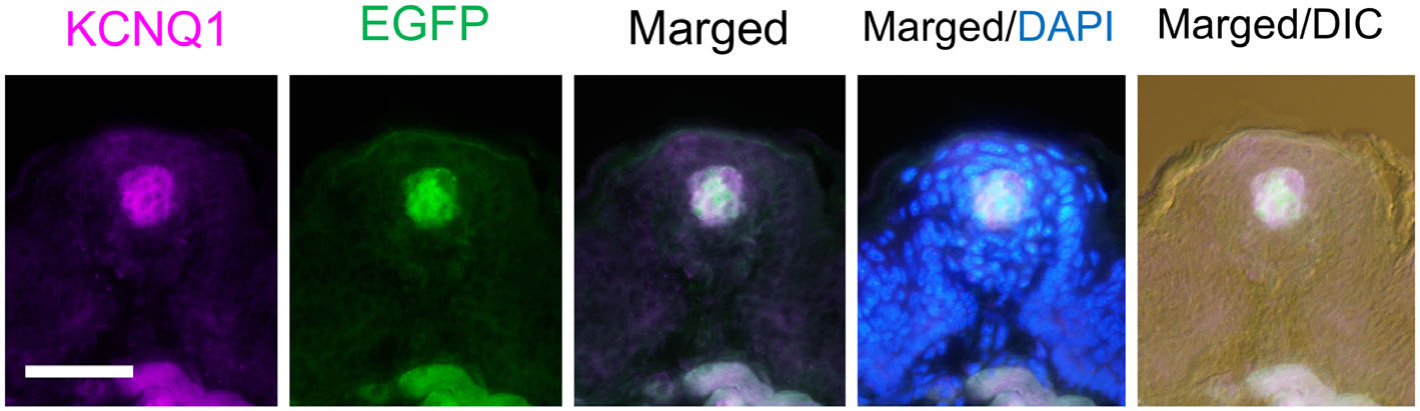
Coexpression of TMC4 (EGFP) and KCNQ1 in the fungiform papillae of *Tmc4*‐EGFP mice. Double immunofluorescent staining was performed using antibodies against KCNQ1 (magenta) and EGFP (green). Merged images also include DAPI nuclear staining (blue). The rightmost panel shows merged images overlaid with differential interference contrast (DIC). Representative image from *n* = 1 biologically independent mouse. Scale bar: 50 μm.

EGFP signals were also detected in the FuP of *Tmc4*‐EGFP mice. Among these, 95.9% of EGFP‐positive cells overlapped with KCNQ1‐positive cells (Fig. 5 and Table 2). These results indicate that TMC4 is expressed in type I, II, and III taste cells in the FuP.

**Table 2:** The coexpression ratio of EGFP and KCNQ1 in the fungiform papillae. Total number of counted cells was given in parentheses.

	Coexpression ratio (%)
EGFP/KCNQ1^a^	95.9% (164/171)
KCNQ1/EGFP^b^	100% (164/164)

^a^ The coexpression ratio of the cells expressing both EGFP and KCNQ1 per the cells expressing KCNQ1

^b^ The coexpression ratio of the cells expressing both EGFP and KCNQ1 per the cells expressing EGFP.

## Discussion

Sodium chloride (NaCl) is a prototypical salty‐tasting compound. While Na^+^ has traditionally been viewed as the primary driver of salt taste, accumulating evidence suggests a contributory role for Cl^−^ as well. In humans, sodium salts with non‐chloride anions (e.g., sulfate or bicarbonate) are perceived as less salty than NaCl, whereas chloride salts like KCl also elicit salty taste, despite an added bitter component [18, 19, 20]. Thus, both Na^+^ and Cl^−^ contribute to salt taste perception.

Salty taste preferences vary depending on the concentration. Rodents prefer NaCl at low concentrations (< 100 mm) but avoid high concentrations (> 300 mm). These preferences are mediated by different gustatory nerves: the chorda tympani nerve transmits low‐concentration signals, while the glossopharyngeal nerve conveys high‐concentration signals [5, 10]. At the molecular level, the low‐salt response is inhibited by the ENaC inhibitor amiloride, defining the AS pathway. In contrast, the high‐salt response persists in the presence of amiloride and is termed the AI pathway.

Recently, it was revealed that TMC4 functions as a voltage‐dependent chloride channel, and deletion of the *Tmc4* gene significantly reduces glossopharyngeal nerve responses to high concentrations of salty stimuli. These findings indicate that TMC4 serves as a key molecule in AI‐mediated salt perception.

To investigate the cellular localization of TMC4, we generated *Tmc4*‐EGFP knock‐in mice by inserting an EGFP reporter downstream of the *Tmc4* stop codon using the CRISPR‐Cas9 system. Due to the lack of specific commercial antibodies against TMC4, we utilized EGFP expression as a surrogate marker to visualize TMC4 localization. Immunostaining confirmed that EGFP signals overlapped with the *Tmc4* mRNA expression pattern, validating the model's utility in identifying TMC4‐expressing cells.

Since the *Tmc4* mRNA signal has been observed to be stronger in the CvP than in the FuP [11], we initially focused our analysis on the CvP. Co‐immunostaining with EGFP and cell‐type‐specific markers confirmed that EGFP‐positive cells colocalize with both type II and type III markers (Gustducin, PLCβ2 and AADC). Moreover, EGFP signals coincided with KCNQ1 signals. In addition, triple staining identified EGFP‐positive cells that did not co‐express type II (PLCβ2) or type III (CAR4) markers, suggesting expression of TMC4 in type I cells. These results demonstrate that TMC4 is expressed across all mature taste cell types: type I, II, and III. We also examined the FuP and observed EGFP signals overlapping with KCNQ1, indicating expression of TMC4 in multiple taste cell types. Our previous study reported that *Tmc4* mRNA colocalizes with markers for type I, II, and III taste cells [11]. The present data obtained from *Tmc4*‐EGFP mice are consistent with these mRNA expression patterns. Furthermore, a recent single‐cell RNA sequencing of cells from the CvP demonstrated that *Tmc4* mRNA is expressed in all three types of taste cells [21]. It is known that type I, II, and III cells make up approximately 50%, 30%, and 20% of taste bud cells, respectively [22]. Consistent with this, the present study also found that PLCβ2‐positive cells accounted for 28.0% and AADC‐positive cells for 16.8%.

It is generally known that Cl^−^ influx through chloride channels contributes to membrane hyperpolarization [23]. From molecular simulation analysis, TMC4 was predicted to shorten the duration of action potentials in response to salt stimulation, thereby increasing firing frequency under sustained exposure. Specifically, TMC4 is hypothesized to accelerate the salt taste signaling cycle by facilitating chloride ion entry into taste cells, thus enabling the generation of new salt taste signals [11]. However, the specific upstream molecules that trigger action potentials remain unidentified.

Regarding the effect of amiloride on salt taste perception in humans, sensory studies have reported that amiloride does not significantly influence salt taste perception [20, 24, 25]. Additionally, cell‐based experiments have shown that the half‐maximal inhibitory concentration of the ENaC δβγ isoform is approximately 25 times higher than that of ENaCαβγ (IC_50_: ENaC δβγ 2.7 μm vs. ENaCαβγ 0.11 μm) [26, 27]. These findings suggest that ENaC δβγ is less sensitive to amiloride, and that the AS salt taste pathway may play only a limited role in human salt taste perception. Therefore, understanding AI pathways is essential for elucidating the mechanisms of salt taste reception in humans.

## Conclusions

In this study, we demonstrated that TMC4 is broadly expressed in all types of mature taste bud cells. Although the molecular mechanisms underlying salty taste perception remain incompletely understood, previous studies have implicated type II cells in the AS pathway [28], and both bitter‐responsive type II and type III cells in aversive responses to high‐salt concentrations [5, 6]. Furthermore, a potential involvement of type I cells in salty taste detection has also been suggested [7]. These findings collectively indicate that all three mature cell types, types I, II, and III, may participate in salty taste detection. Our finding that TMC4 is expressed in type I, II, and III cells suggests that it may contribute to multiple aspects of AI salty taste transduction, rather than being restricted to a single‐cell type. Future investigations into the cell‐type‐specific roles of TMC4, as well as its molecular interaction partners, will be crucial for elucidating the cellular and molecular basis of AI salty taste transduction.

## Conflict of interest

The authors declare no conflict of interest.

## Author contributions

TA and MN designed the overall research concept. MM, YS, and MN collected and analyzed the data. YK, WF, and TM interpreted the data. MN wrote the manuscript. All authors contributed to writing – review and editing.

## Supporting information


**Fig. S1.** Fluorescence reporter EGFP expression in the circumvallate papillae.

**Fig. S2.** Negative control for *in situ* hybridization using a *Tmc4* sense probe in the circumvallate papillae.

**Fig. S3.** Negative control staining in the circumvallate (CvP) and fungiform papillae (FuP).

**Table S1.** Primers for genotyping.

## Data Availability

The data that support the findings of this study are available from the Dryad Digital Repository: https://doi.org/10.5061/dryad.sbcc2frks.
