# Supplementary figures and images for: TMC4 localizes to multiple taste cell types in the mouse taste papillae
Source: FEBS Open Bio. 2025 Nov 11;16(4):778–87. doi: 10.1002/2211-5463.70159 (PMC13042416; doi:10.1002/2211-5463.70159)

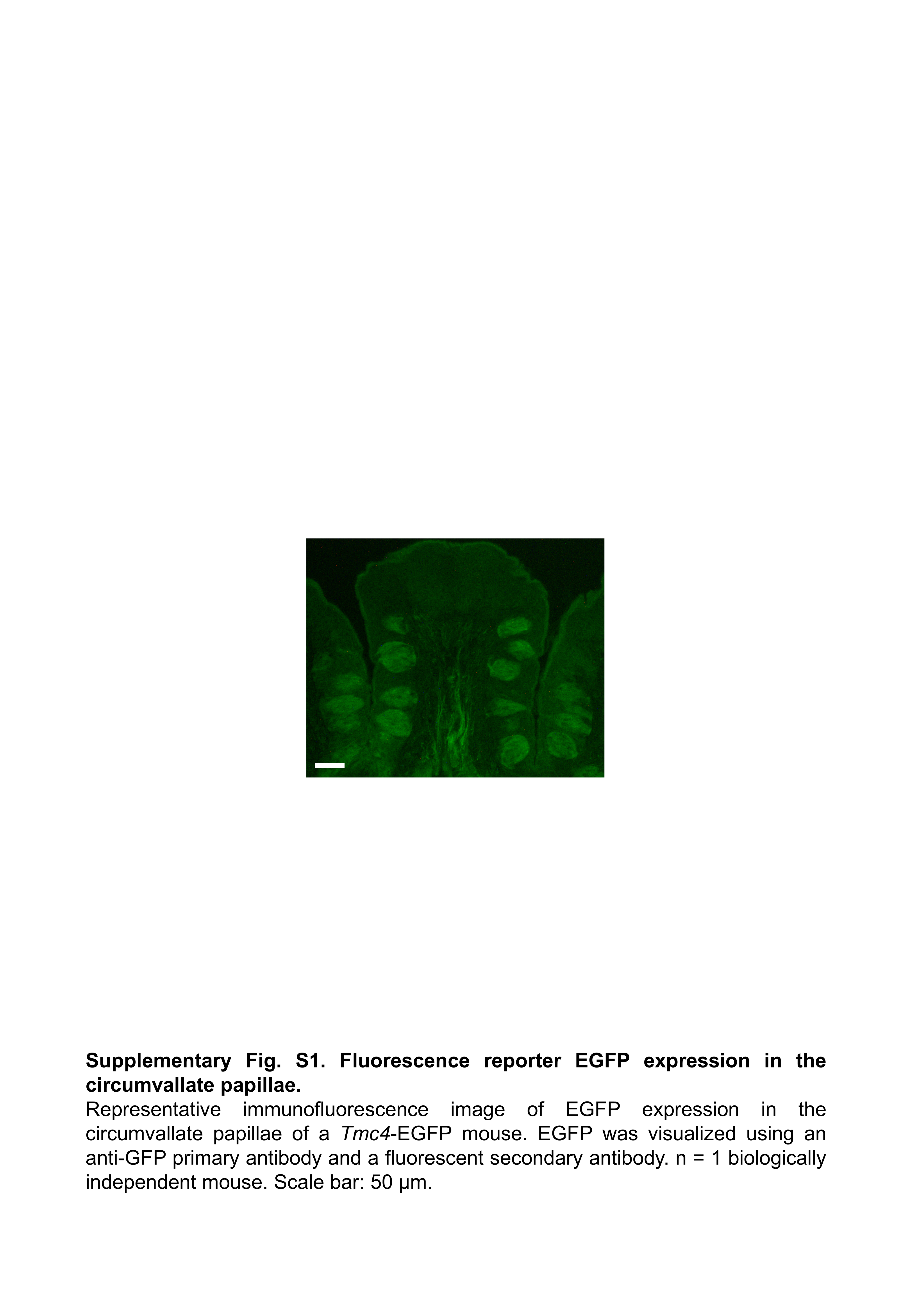

Supplement: Supplementary file 1 — Fig. S1. Fluorescence reporter EGFP expression in the circumvallate papillae. [file FEB4-16-778-s002.tiff]

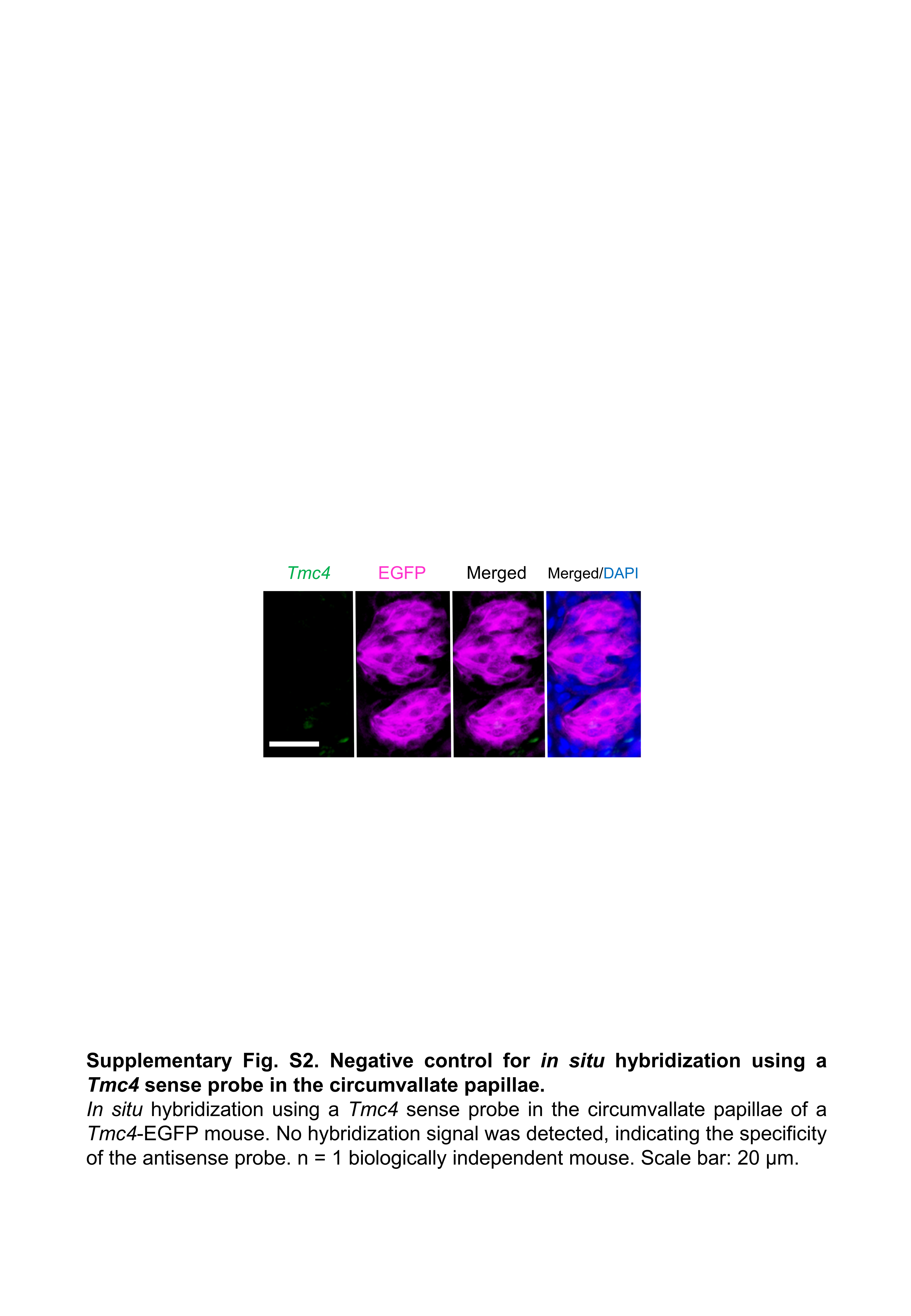

Supplement: Supplementary file 2 — Fig. S2. Negative control for in situ hybridization using a Tmc4 sense probe in the circumvallate papillae. [file FEB4-16-778-s004.tiff]

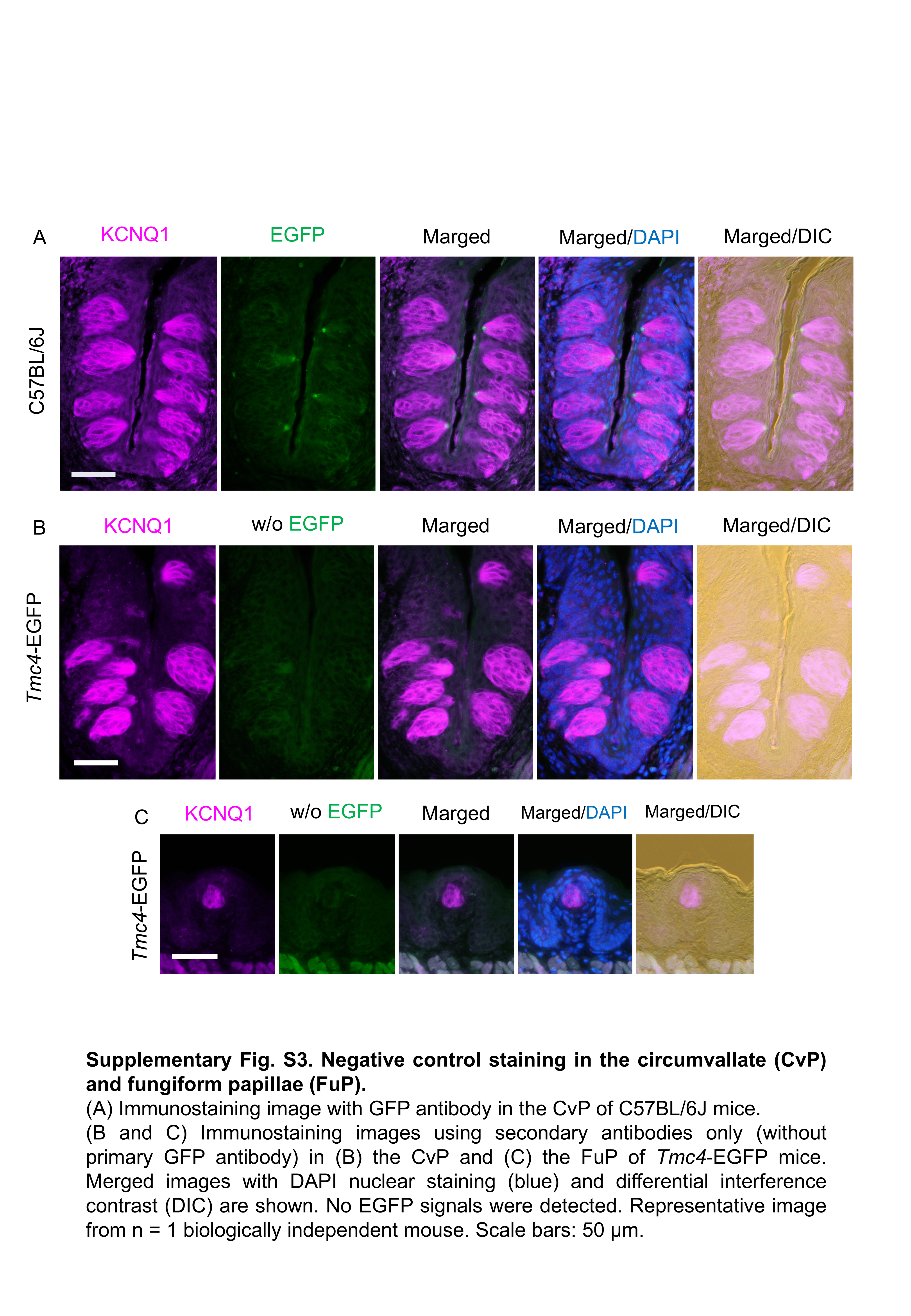

Supplement: Supplementary file 3 — Fig. S3. Negative control staining in the circumvallate (CvP) and fungiform papillae (FuP). [file FEB4-16-778-s001.tiff]
