# Supplementary material for: TMC4 localizes to multiple taste cell types in the mouse taste papillae
Source: FEBS Open Bio. 2025 Nov 11;16(4):778–87. doi: 10.1002/2211-5463.70159 (PMC13042416; doi:10.1002/2211-5463.70159)
Supplement: Supplementary file 4 — Table S1. Primers for genotyping. [file FEB4-16-778-s003.pdf]

Supplementary Table S1. Primers for genotyping.

Primers	Sequences	Product size (bp) WT	HT	KI
Tmc4 KI F	AACTGTTACGGACGCCTCATC			
Tmc4 KI R	TGTAACCTGAGTTGCTCCACC	516	516	
GFP R	CTGCTTGTCGGCCATGATATAGAC		1325	1325
